# Changes in Species Richness and Composition of Tiger Moths (Lepidoptera: Erebidae: Arctiinae) among Three Neotropical Ecoregions

**DOI:** 10.1371/journal.pone.0162661

**Published:** 2016-09-28

**Authors:** Hernán Mario Beccacece, Sebastián Rodolfo Zeballos, Adriana Inés Zapata

**Affiliations:** 1 Centro de Investigaciones Entomológicas de Córdoba, Instituto de Investigaciones Biológicas y Tecnológicas, Consejo Nacional de Investigaciones Científicas y Técnicas- Universidad Nacional de Córdoba, Córdoba, Argentina; 2 Instituto Multidisciplinario de Biología Vegetal, Consejo Nacional de Investigaciones Científicas y Técnicas-Universidad Nacional de Córdoba, Córdoba, Argentina; 3 Grupo de Investigación y Conservación de Lepidópteros de Argentina, Museo de Zoología, Facultad de Ciencias Exactas, Físicas y Naturales, Universidad Nacional de Córdoba, Córdoba, Argentina; University of Sydney, AUSTRALIA

## Abstract

Paraná, Yungas and Chaco Serrano ecoregions are among the most species-rich terrestrial habitats at higher latitude. However, the information for tiger moths, one of the most speciose groups of moths, is unknown in these ecoregions. In this study, we assess their species richness and composition in all three of these ecoregions. Also we investigated whether the species composition of tiger moths is influenced by climatic factors and altitude. Tiger moth species were obtained with samples from 71 sites using standardized protocols (21 sites were in Yungas, 19 in Paraná and 31 in Chaco Serrano). Rarefaction-extrapolation curves, non-parametric estimators for incidence and sample coverage indices were performed to assess species richness in the ecoregions studied. Non metric multidimensional scaling and *adonis* tests were performed to compare the species composition of tiger moths among ecoregions. Permutest analysis and Pearson correlation were used to evaluate the relationship among species composition and annual mean temperature, annual temperature range, annual precipitation, precipitation seasonality and altitude. Among ecoregions Paraná was the richest with 125 species, followed by Yungas with 63 species and Chaco Serrano with 24 species. Species composition differed among these ecoregions, although Yungas and Chaco Serrano were more similar than Paraná. Species composition was significantly influenced by climatic factors and altitude. This study showed that species richness and species composition of tiger moths differed among the three ecoregions assessed. Furthermore, not only climatic factors and altitude influence the species composition of tiger moths among ecoregions, but also climatic seasonality at higher latitude in Neotropical South America becomes an important factor.

## Introduction

Knowledge of species’ distributions is fundamental not only to understand the ecological and evolutionary determinants of spatial patterns of biodiversity [1–3], but also to develop better conservation planning and forecasting [4,5]. It is well known that several factors influence the distribution of species such as climate, geographical barriers, land use, species characteristics and biotic interactions [6–8]. Therefore, assessing the effects of those factors that determine species’ distributions is not only of theoretical interest, but also a key to understanding species responses to environmental changes [6,9].

Under a climate change and habitat loss scenario, insects are a good model for researchers who attempt to understand actual species distribution patterns [10] because of their rapid response to environmental changes [11]. Neotropical South American is one of the most species-rich regions in the world, where insects represent more than 80% of all known worldwide insects. This region also shows a high level of endemism [12]. Among insects, the tiger moths (Lepidoptera: Erebidae: Arctiinae) are one of the most speciose groups with almost 11,000 species worldwide, of which at least half are present in the Neotropical region [13,14]. This group of moths has been frequently used as a model for diversity studies in Neotropical South America, with analyses of distribution patterns in response to habitat heterogeneity, altitudinal gradient and climatic factors [15–20]. Nevertheless, almost all of these studies are focused on comparisons of the species richness and composition of tiger moths at regional-scales, highlighting the lack of larger, macro-scale studies (i.e., those that encompass more than one ecoregion). Such studies are necessary to allow us to fully understand which factors constrain the distribution and diversity of tropical and subtropical insects globally [20,21].

The diversity and distribution of species interact in space and time at different spatial scales [22]. Climatic conditions (e.g., temperature and precipitation), are some of the main factors that contribute to patterns of species distribution among regions [8,22,23]. Furthermore, those factors that determine strong physical changes (e.g., altitude) become more important determining the assemblage of species from those available in the regional pool [24–26]. In Neotropical South America it has been observed that species richness and composition of tiger moths were related to climatic factors as well as to altitude [15,16,20,27,28]. Fiedler et al. [29] and Zenker et al. [20] observed changes in the species richness and composition of tiger moths along an altitudinal gradient in mountains of the Andes and Serra do Mar respectively. Along an altitudinal gradient, changes in temperature and availability of resources occur sharply with a strong influence on biodiversity patterns [30]. Furthermore, Ferro & Melo [16] working in the same Brazilian Atlantic forest as Zenker et al [20] but at a broader-scale, observed that variation in species composition was mainly related to changes in annual mean temperature, temperature range and altitude changes. No study has tested how climatic factors affect the diversity of tiger moths among ecoregions present at higher latitude in Neotropical South America where climate plays an important role in the distribution of terrestrial species [23,31].

At higher latitudes in Neotropical South America, the Yungas and Paraná are two ecoregions considered as biodiversity hotspots [12] and, jointly with Chaco Serrano, are some of the most important ecoregions with the highest species richness [32,33]. Despite this, there is a surprising absence of information of the tiger moth fauna. The only current information available is a checklist from Chaco Serrano [34]. Due to their proximity and difference in climatic conditions and altitude, these ecoregions are areas of interest to test the effect of these variables on the tiger moth fauna. Therefore, we aim to determine the species richness and composition of tiger moths from the Yungas, Paraná and Chaco Serrano ecoregions of Neotropical Argentina and how diversity is influenced by climatic factors and altitude. Specifically, we assess 1) the species richness of tiger moths in each ecoregion; 2) if the species composition changes among these three ecoregions; and 3) if the species composition is related to climatic factors (temperature annual range, annual mean temperature, annual precipitation and precipitation seasonality) and altitude.

## Materials and Methods

### Study Area

**Fig 1:**
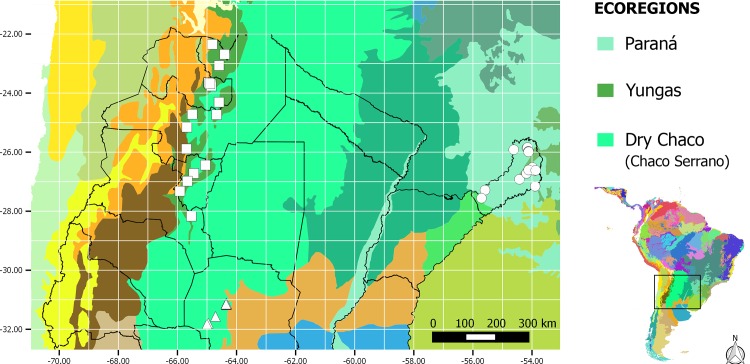
Location of the sampled sites. The map was constructed based on the WWF terrestrial ecoregion maps (World Wildlife Fund). Each symbol represents a sampled site: Circle = Paraná; Square = Yungas; Triangle = Chaco Serrano.

The study was carried out in three ecoregions across the north-east, north-west and the center of Argentina: Yungas, Paraná and Chaco Serrano (Fig 1 and S1 Table). Maps of sampling sites were made using QGIS 2.6.14 based on the World Wildlife Fund terrestrial ecoregion maps [35]. The Southern Andean Yungas, also called “Yungas”, extends from the eastern slopes of the Andes in northern Peru to northwestern Argentina [33]. In Argentina, it is located between 400 and 3000 m a.s.l., and it has a subtropical climate, with a dry season. The total annual rainfall is around 900–2000 mm, the mean temperature of the warmest month is approximately 22°C and the mean annual temperature is approximately 18°C with a cold season during April-November [33,36]. The Alto Paraná Atlantic forest, or “Paraná”, is an ecoregion located in southeastern Brazil, northeastern Argentina and eastern Paraguay. In Argentina, it is located between 150 and 900 m a.s.l., with a subtropical humid climate and no dry season. The total annual rainfall is around 1000–2200 mm and its mean temperature ranges from 16–22°C with a cold season during June-August [33,37,38]. The Chaco Serrano dry subtropical forest, or “Chaco Serrano”, belongs to the western dry Chaco ecoregion, it is located in the center of Argentina, between 500 and 1300 m a.s.l., with a temperate, semi-arid climate. It has a monsoonal precipitation regime with an average annual rainfall of 750 mm, mainly concentrated in the warm season (October–April). Its mean temperature ranges from 10°C to 26°C [33,39]. In this study, we sampled the southern sites of native Chaco Serrano forest that are located in Córdoba province, because most of this ecoregion has been lost to anthropogenic fragmentation [39].

### Ethics statement

Capture of specimens and their transport to the laboratory for subsequent identification were permitted by Administración de Parques Nacionales (Yungas), Ministerio de Ecología y RNR (Paraná) and Agencia Córdoba Ambiente (Chaco Serrano).

### Sampling Methodology and Taxonomic Identification

Tiger moths were sampled at 71 sites: 21 in Yungas, 19 in Paraná and 31 in Chaco Serrano. The samples were made during the new moon phase and without rain in the warm seasons from 2007 to 2013 over periods that encompassed an average of 5 hours, beginning in the late afternoon and ending when not one new species or morphospecies was collected over 1 hour [15]. Each sample site was selected to represent the typical plant community and landscape of the ecoregion.

Tiger moths were attracted using a light trap, which consisted of a white 2 × 2 m sheet stretched over a frame, and illuminated with a 250 w mercury, mixed light lamp (Osram HWL model with a spectral power distribution at visible spectrum ranges, https://www.osram.com). Specimens were collected manually and sacrificed in killing jars with ethyl acetate. Then, in the laboratory specimens were mounted, labeled and identified into species or morphospecies. The identifications were made mainly with genitalia dissections, comparison with type material and consultations with specialists (see acknowledgments). Specific and general classification was made following Vincent & Laguerre [14]. The material was deposited in the Museo de Zoología, Universidad Nacional de Córdoba (MZUC), in the Grupo de Investigación y Conservación de Lepidópteros de Argentina (GICLA) collection. As in other studies, almost 30% of the specimens collected are described as morphospecies [20,27] and the majority belong to the subtribe Lithosiina. The morphospecies ‘problem’ illustrates the lack of taxonomic studies of tiger moths. However, voucher specimens are available for further studies.

### Data Analysis

In order to evaluate if the tiger moth species from the three ecoregions were well represented and could be compared, incidence-based rarefaction and extrapolation (R/E) curves were performed by two methodologies: sample-size- and coverage-based. These methodologies represent a unified sampling framework from which to make fair and meaningful comparisons of species richness among multiple assemblages [40]. The sample-size R/E approach compares diversity estimates with respect to sample size while the coverage-based R/E curve compares species richness of a set of assemblages based on samples of equal completeness (equal coverage) [41]. As suggested by Chao et al. [41], the rarefaction curves were extrapolated to double the number of sampling sites of the ecoregion with the lowest sampling sites (Paraná with 19), but considering the number of sampling sites of the ecoregion with the highest sampling sites (Chaco Serrano with 31). Therefore, the extrapolation was extended to a sample size of 31, configured at 40 knots and 100 replicate bootstrapping runs to estimate 95% confidence intervals [42–44]. The curves and the sample coverage were performed with iNext software [44]. In addition, four non-parametric estimators for incidence data (Chao2, ICE, Jackknife 1, Jackknife 2) were used to estimate the total number of species that would be present in each ecoregion. These non-parametric estimators correct the observed species by adding a term based on the frequencies of species represented in only one (unique) sample, in two (duplicates), or in a few replicate incidence samples [45]. Non-parametric estimators for incidence data were performed with EstimateS version 9.1.0 software [46].

Non-metric Multidimensional Scaling (NMDS) analysis was used to compare the composition of tiger moths among the three ecoregions. NMDS was performed using species presence/absence per site matrix and the Chao-Jaccard similarity index. This index includes the effect of unseen shared species, based on replicated incidence-based sample data, and proves to be considerably less biased than classic indices when a substantial proportion of species are missing from samples [47]. We employed a Permutational Multivariate Analysis of Variance (PERMANOVA) to determine whether there were differences in the species composition among the three ecoregion. We used *adonis*, with distance Chao-Jaccard similarity index and 9999 random permutations [48]. *Adonis* partitions dissimilarities (typically based on species-in-sites data) for the sources of variation, and uses permutation tests to inspect the significances of those partitions [48]. We also analyzed pairwise differences among ecoregions.

The relationship between species composition and both variables, climatic factors and altitude, was tested through a Permutest analysis [48]. Furthermore, Pearson correlation analysis was performed between the first two NMDS axes and climatic factors and altitude, to analyze the relative effect of these variables on the tiger moth species composition. Climatic factors were compiled from the Worldclim database (www.worldclim.org) [49], at a spatial resolution of 2.5 arc-minutes for continental South America. Altitude was registered at each sampling site. Four climatic factors that did not show colinearity among them were selected (r<0.75): annual mean temperature, annual precipitation, precipitation seasonality and annual temperature range. These factors corresponded to average conditions for the time period 1950–2000 [49]. All mapping and data extraction was conducted using QGIS 2.6.14 [35]. The Chao-Jaccard index, NMDS ordination, Adonis, Permutest and Pearson correlation were done with R using the package `vegan´ and `mass´ 2.0–6 [48,50].

## Results

**Fig 2:**
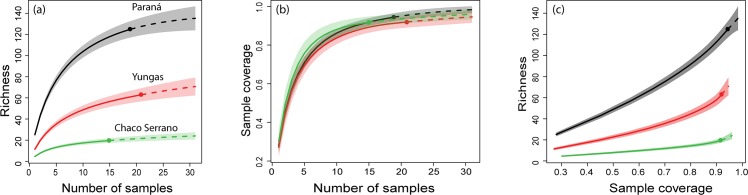
**Rarefaction and Extrapolation Sample-size-based rarefaction and extrapolation (a), sample completeness curve (b) and coverage-based rarefaction (c) for each ecoregion (Paraná, Yungas and Chaco Serrano).** In all figures, solid lines of the curves represents rarefaction, the dashed lines represents extrapolation (up to a maximum sample size of 31) and shaded areas the 95% confidence intervals (based on a bootstrap method with 100 replications).

The observed species richness showed that Paraná is the richest ecoregion (125 species), followed by Yungas (63) and Chaco Serrano (24) (S2 Table). The incidence-based rarefaction curves showed that the asymptote was not very far from being reached for any of the three ecoregions, while the extrapolated curves showed that Paraná is the richest ecoregion analyzed, follow by Yungas and Chaco Serrano (Fig 2A) as in the observed species richness. The estimated sample coverage suggested that richness at all ecoregion studied was well represented with a sample coverage percentage in excess of 90% (Fig 2B and 2C, S1 and S3 Tables). On the other hand, the observed species richness in each ecoregion reached an important percentage of the species estimated using the non-parametric estimators (S3 Table). The total number of species observed in Paraná represent between 80.13% and 89.93%, in Yungas 67.74% and 77.78% and in Chaco Serrano 75% and 88.89% of expected species respectively.

**Fig 3:**
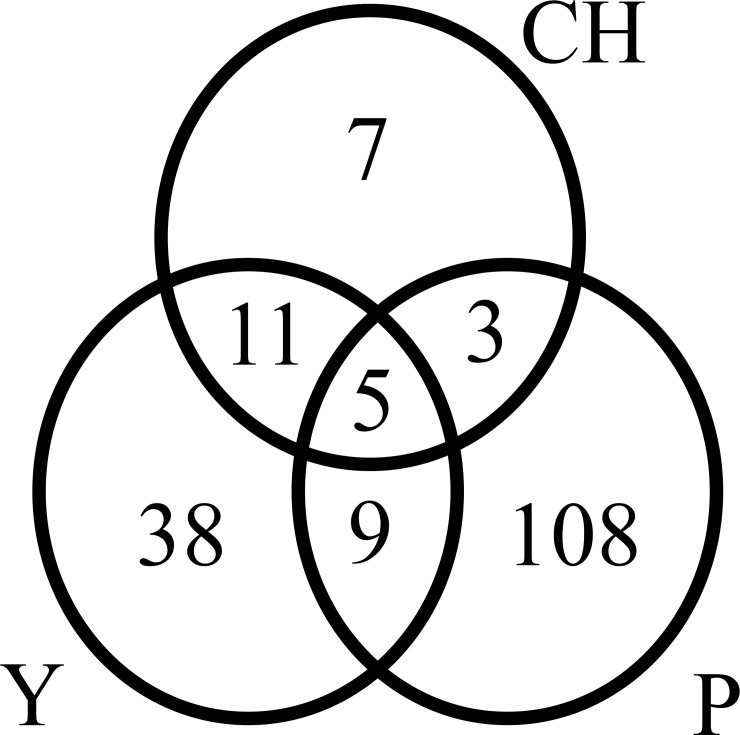
Venn diagram of species richness among the three ecoregions. Each circle represents an ecoregion, Y: Yungas, P: Paraná and Ch: Chaco Serrano. Numbers indicate the species found exclusively in each habitat, in two ecoregions or in all of them.

When the species composition was compared in each region, 108 were exclusive to Paraná, 38 to Yungas and six to Chaco Serrano. Paraná and Yungas shared 10 species whilst Paraná-Chaco Serrano and Yungas-Chaco Serrano shared 3 and 11 species respectively. Finally, only 4 species were present in all ecoregions (Fig 3 and S2 Table).

**Fig 4:**
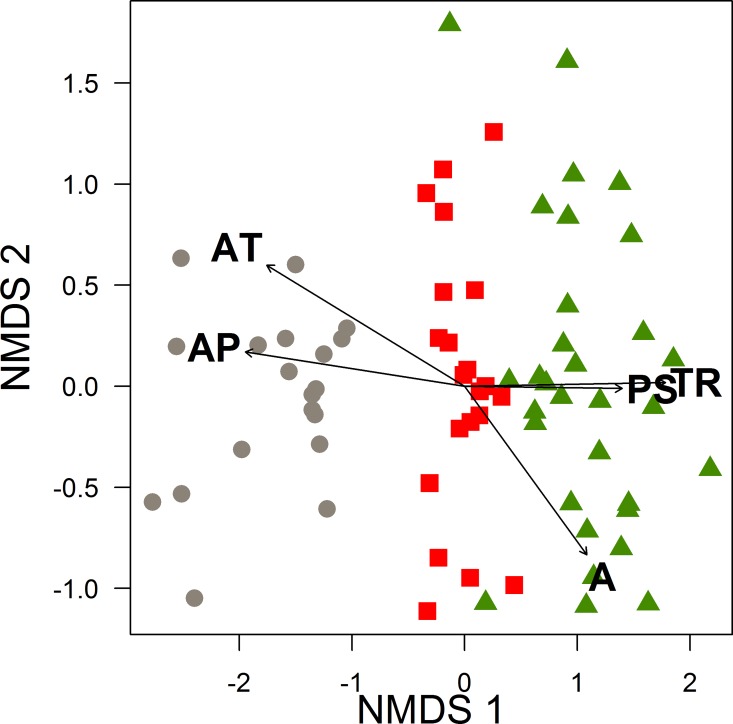
Ordination plot with the fitted surface of the four significant climatic factors using NMDS of presence/absence data. A 71 sites × 180 tiger moth species matrix was used for the three ecoregions. Stress values were 0.13 of this data set. Abbreviations: A = altitude; AT = annual mean temperature; TR = annual range temperature; AP = annual precipitation; PS = precipitation seasonality. Grey circles = Paraná, red squares = Yungas, and green triangle = Chaco Serrano. Each symbol represents one sampled site.

Along axis 1 of the NMDS, consistent differences among the three areas were observed (Fig 4). Paraná sites scored in the left-hand side of the ordination, while on the right-hand side were the Chaco Serrano sites (Fig 4). In reduced ordination space, tiger moth richness obtained at Yungas sites appeared intermediate between those from Paraná and Chaco Serrano sites. However, Yungas sites appeared more similar to the Chaco Serrano sites (Fig 4). Furthermore, the species composition among the ecoregions differed significantly (Adonis, F = 12.608, p = 0.0001; S3 Table).

The composition of tiger moth communities was significantly related to annual precipitation (r^2^ = 0.71; p = 0.001), annual mean temperature (r^2^ = 0.60; p = 0.001), annual temperature range (r^2^ = 0.58; p = 0.001), precipitation seasonality (r^2^ = 0.41; p = 0.001) and altitude (r^2^ = 0.42; p = 0.001). Paraná sites showed the highest annual mean temperature and annual precipitation, the lowest precipitation seasonality and annual temperature range (Fig 4). Conversely, Chaco Serrano sites showed the highest precipitation seasonality, annual temperature range and showed the lowest annual mean temperature and annual precipitation (Fig 4). Furthermore, NMDS axis 1 was related mainly to annual precipitation (r = 0.84; p = 0.001) followed by annual mean temperature (r = -0.77; p = 0.001), annual temperature range (r = 0.76; p = 0.001), precipitation seasonality (r = 0.61; p = 0.001) and altitude (r = 0.54; p = 0.001), while only altitude was related to axis 2 (r = -0.26; p = 0.03).

## Discussion

Almost all previous studies of tiger moths in Neotropical South America were made at low latitude and focused on describing the species richness and composition and their responses to biotic and abiotic factors at a regional or local-scale [15–19,27–29,51–56], with some exceptions that encompass more than one ecoregion [20,57]. Furthermore, as far as we know no study until now has tested how the species richness and composition of tiger moths from Neotropical South America varies among regions at higher latitudes. Our study not only describes and compares unknown species compositions from three ecoregions, but also how the species composition is related to climatic factors and altitude.

In spite of there being some exceptions [58], diversity studies of Lepidoptera in Neotropical South America often report richness decreases with increasing latitude [1,57,59–61]. One explanation for this pattern is that at higher latitudes climate becomes more important in constraining the distributions of species, especially for ectothermic animals [26,57,62,63]. As in other Lepidoptera, a similar richness decrease pattern can be observed in tiger moths species. For instance, at lower latitude in montane rainforest from southern Ecuador with a more stable climate during the year, 415 species were recorded (Chao2 = 588.95) [64]. At middle latitude in the Brazilian Cerrado with a seasonal tropical climate, 197 tiger moth species (Chao2 = 383.8 species) were found [52]. At higher latitude, in the southern Atlantic Forest of Brazil, at a similar latitude to our Yungas and Paraná sampling sites, 121 species (Chao2 = 162 species) were found [17]. In our study, Paraná at the lowest latitude had 125 species (Chao2 = 138 species), followed by Yungas at similar latitude to Paraná with 63 species (Chao2 = 83.11 species) and Chaco Serrano at the highest latitude with 24 species (Chao2 = 26.9 species) (S1 Table). Nonetheless, despite the fact that Yungas and Paraná occur at similar latitudes, these ecoregions showed a marked difference in their species richnesses. This difference could be related to the proximity between Paraná and Serra do Mar (Chao2 = 319 species), an important hotspot of tiger moths in the Neotropical region [20]. Moreover, beyond the neotropics in South America, the high latitude Magellanic subpolar forest ecoregion includes only 3 tiger moth species [65]. Thus the decrease in species richness of tiger moths with increasing latitude suggests that the different climatic conditions among ecoregions might have an effect over species distribution [31].

At a macro-scale the evidence suggests that species composition is affected mainly by environmental factors, physiographic characteristics and historical events [8,66,67]. In this sense, we observed that the species composition of tiger moths was related not only to annual mean temperature, annual mean precipitation and annual temperature range, as Ferro & Melo [16] observed in Atlantic forest, but also to precipitation seasonality. Nevertheless, the species composition seems to be affected by climatic factors differently in each ecoregion. The species composition in Paraná was associated with a more stable climate determined by the highest annual mean temperature and annual precipitation, while Yungas and Chaco Serrano were associated with a seasonal climate with the lowest annual mean temperature and precipitation. Our results (Fig 4, Permutest and Pearson correlation) show that the climatic seasonality becomes an important factor in the distribution of tiger moths at higher latitudes. Nonetheless, some authors suggest that vegetation types also influence significantly the assemblages of tiger moths [17,19,27,28,51,52,55]. Although we could not study the vegetation type, the resulting NMDS ordination indirectly reflected strong vegetation units that encompass plant communities from subtropical rainforest in Paraná, montane forest in Yungas to dry forest in Chaco Serrano. Future studies must take into account the vegetation types to fully understand those factors that structure tiger moth distribution patterns.

Previous studies have also observed that the species composition of some groups of Lepidoptera (including tiger moths), was affected by altitude [20,29,62,68]. Similarly, we observed that the variability in species compositions of tiger moths from the three ecoregions seems to be related to altitudinal gradients. Zenker et al [20] and Brehm et al [68] suggest that along an altitudinal gradient there were changes in species composition related to changes in climate and vegetation. Furthermore, Brehm [57] suggests that the thermal biology of species could be limiting the distributional patterns of tiger moths over an altitudinal gradient, because temperature strongly affects their flight. Therefore, our results and previous studies suggest that altitudinal zonation exists within an ecoregion, where some species of tiger moths cannot cover a wide area having restricted distributional ranges [16,20].

The species richness and composition differences among ecoregions are linked not only to present-day climatic conditions, but also to historical processes acting at evolutionary and ecological time-scales [67,69]. In this sense, two complementary hypotheses could allow us to explain why the species composition of Yungas is in between Chaco and Paraná, and also why Paraná and Yungas are more dissimilar. During the climatic fluctuations of the Pleistocene, Yungas, Paraná and Chaco were merged due to climatic vegetational fluctuations and some modern remnant patches that act as relict connections, allowing some species to expand their distributional ranges [70]. This hypothesis is used to explain the distribution pattern of some marsupials, birds and harvestmen [71–73]. On the other hand, the spatial proximity of Yungas to Chaco habitats could explain this closer association. Ferro & Romanowski [17] suggest that the spatial proximity among sampled sites could explain changes in the assemblages of tiger moths. Therefore, the actual climate, in addition to historical contingencies and physiographic conditions, seem to work together, modulating species compositions and species distributions among and within each ecoregion.

Our study adds evidence to what we know about the influence of climatic factors on the distribution of tiger moths [16,20]. Furthermore, in the absence of vegetation data, our analysis could give insight into the scaling hierarchy of regional vs. macro-scale influences of climatic factors and altitude in shaping moth assemblages. At a macro-scale climatic factors seemed to exert the main constraint over the distribution of tiger moths among ecoregions, while at a regional-scale (within each ecoregion) altitude seemed to affect the species composition of tiger moths. Therefore, more research focused on targeted study organisms, such as tiger moths at different spatial scales, should help to achieve better understanding of global species distribution patterns and the influence of environmental factors. It is especially necessary to conduct field studies using standardized sampling methodology across multiple ecoregions [74].

## Supporting Information

S1 Table Detailed information of sampling sites. Information of 71 sampling sites (province from Argentina, date, geographic location, altitude and biogeographical area), number of species observed and sample coverage for each sampling site. (DOCX)
(DOCX)

S2 Table List of tiger moth species. Presence/Absence of the tiger moth species in each ecoregion. (DOCX)
(DOCX)

S3 Table Species observed and non-parametric estimators for incidence data in each ecoregion. (DOCX)
(DOCX)

S4 Table A*donis* test partitioning variation on species composition. Evaluated by Chao-Jaccard distance matrix and pairwise comparison between ecoregions. (DOCX)
(DOCX)
