# Supplementary material for: Changes in Species Richness and Composition of Tiger Moths (Lepidoptera: Erebidae: Arctiinae) among Three Neotropical Ecoregions
Source: PLoS One. 2016 Sep 28;11(9):e0162661. doi: 10.1371/journal.pone.0162661 (PMC5040457; doi:10.1371/journal.pone.0162661)
Supplement: S1 Table — Information of 71 sampling sites (province from Argentina, date, geographic location, altitude and biogeographical area), number of species observed and sample coverage for each sampling site. (DOCX) (DOCX) [file pone.0162661.s001.docx]

Hernán M. Beccacece, Sebastián R. Zeballos and Adriana I. Zapata

Changes in species richness and composition of tiger moths (Lepidoptera: Erebidae: Arctiinae) among three neotropical ecoregions

PLOS ONE

**Table S1 Detailed information of sampling sites.** Information of 71 sampling sites (province from Argentina, date, geographic location, altitude and biogeographical area), number of species observed and sample coverage for each sampling site. PP = Provincial park, PN = National Park, R = Reserve, RP = Private Reserve, Y = Yungas, P = Parana, CH = Chaco Serrano (each site with the code).

| Sampling site | Province | Date | Latitude | Longitude | Altitude  masl. | Ecoregion | Observed species | Sample coverage index |
| --- | --- | --- | --- | --- | --- | --- | --- | --- |
| Campo Quijano | Salta | 16/04/2010 | -65.69611 | -25.88779 | 1662 | Yungas | 5 | 0.271 |
| Quebrada de Escoipe | Salta | 17/04/2010 | -65.68139 | -25.15472 | 1641 | Yungas | 8 | 0.446 |
| R San Lorenzo | Salta | 19/04/2010 | -65.50000 | -24.71667 | 1500 | Yungas | 7 | 0.561 |
| Dique Alijillán | Catamarca | 21/11/2010 | -65.52694 | -28.17056 | 608 | Yungas | 9 | 0.640 |
| El Siambón | Tucumán | 23/11/2010 | -65.44528 | -26.72000 | 1132 | Yungas | 6 | 0.696 |
| Río Cochuna | Tucumán | 24/11/2010 | -65.91611 | -27.32250 | 1099 | Yungas | 18 | 0.738 |
| Camino a San Francisco | Jujuy | 22/11/2010 | -64.94677 | -23.64067 | 1364 | Yungas | 14 | 0.771 |
| PN Calilegua – 1km Mes. Las Colmenas | Jujuy | 25/10/2011 | -64.90664 | -23.69896 | 1272 | Yungas | 17 | 0.797 |
| Rio Nio | Tucumán | 17/12/2011 | -65.05199 | -26.43892 | 1300 | Yungas | 13 | 0.819 |
| PN Calilegua – Mesada Las Colmenas | Jujuy | 26/11/2013 | -64.86742 | -23.69972 | 1135 | Yungas | 22 | 0.837 |
| Camino a San Francisco –1.1km de monolito | Jujuy | 27/11/2013 | -64.89889 | -23.67575 | 1693 | Yungas | 17 | 0.852 |
| Camino a San Francisco – Río Jordán | Jujuy | 28/11/2013 | -64.93725 | -23.64850 | 1415 | Yungas | 18 | 0.865 |
| PN Calilegua – Cerca de Mes. Las Colmenas | Jujuy | 29/11/2013 | -64.85975 | -23.69992 | 1028 | Yungas | 13 | 0.875 |
| PN Calilegua – Mirador | Jujuy | 30/11/2013 | -64.85408 | -23.75825 | 721 | Yungas | 8 | 0.885 |
| PN Calilegua – Cerca de Monolito | Jujuy | 01/12/2013 | -64.90017 | -23.68225 | 1723 | Yungas | 14 | 0.892 |
| Camino a Angosto del Pescado | Salta | 02/12/2013 | -64.40275 | -22.68478 | 467 | Yungas | 6 | 0.899 |
| Camino a Acambuco | Salta | 03/12/2013 | -64.81781 | -22.34614 | 855 | Yungas | 10 | 0.905 |
| Camino a Isla de Caña – O de Ruta Nac. 50 | Salta | 04/12/2013 | -64.59161 | -23.06684 | 547 | Yungas | 10 | 0.909 |
| Camino a Santa Clara – Sierra Santa Bárbara | Salta | 05/12/2013 | -64.59161 | -24.30844 | 1405 | Yungas | 12 | 0.909 |
| PN El Rey | Salta | 06/12/2013 | -64.67328 | -24.72942 | 1002 | Yungas | 6 | 0.917 |
| El Sunchal | Tucumán | 08/12/2013 | -65.66347 | -26.98359 | 1645 | Yungas | 7 | 0.920 |
| PP Teyú Cuaré | Misiones | 18/10/2010 | -55.58835 | -27.28463 | 181 | Paraná | 7 | 0.279 |
| PP Urugua-í | Misiones | 19/10/2010 | -54.16848 | -25.85835 | 286 | Paraná | 3 | 0.453 |
| PP Urugua-í – 4km. al E de Arroyo Uruzú | Misiones | 20/10/2010 | -54.14997 | -25.84125 | 322 | Paraná | 5 | 0.569 |
| PP Cruce de Caballero | Misiones | 21/10/2010 | -53.98667 | -26.52000 | 610 | Paraná | 17 | 0.650 |
| INTA San Vicente – Cuartel Victoria | Misiones | 22/10/2010 | -54.42410 | -26.91910 | 514 | Paraná | 6 | 0.710 |
| PP Cañadón de Profundidad | Misiones | 23/10/2010 | -55.70909 | -27.55844 | 155 | Paraná | 10 | 0.756 |
| PP Esmeralda | Misiones | 17/01/2012 | -53.89194 | -27.15472 | 505 | Paraná | 54 | 0.792 |
| PP Moconá | Misiones | 19/01/2012 | -54.10157 | -26.62028 | 433 | Paraná | 13 | 0.820 |
| San Pedro | Misiones | 27/10/2012 | -54.18665 | -26.71953 | 576 | Paraná | 24 | 0.843 |
| El Paraiso - Camino a Arroyo Fortaleza | Misiones | 28/10/2012 | -54.18667 | -26.71944 | 603 | Paraná | 38 | 0.862 |
| Arroyo Fortaleza | Misiones | 29/10/2012 | -53.92264 | -26.62961 | 423 | Paraná | 40 | 0.877 |
| Ruta 27 – 19km - NE San Pedro | Misiones | 30/10/2012 | -54.11227 | -26.60318 | 586 | Paraná | 46 | 0.890 |
| Camping Fariluk - San Pedro | Misiones | 31/10/2012 | -53.87214 | -26.61431 | 561 | Paraná | 21 | 0.901 |
| Ruta 27 – 36.3km - NE San Pedro | Misiones | 01/11/2012 | -54.61659 | -25.91557 | 585 | Paraná | 41 | 0.911 |
| Puerto Bemberg | Misiones | 05/11/2013 | -54.11659 | -25.97596 | 162 | Paraná | 31 | 0.919 |
| RP Urugua-í | Misiones | 06/11/2013 | -54.11659 | -25.97421 | 277 | Paraná | 40 | 0.926 |
| RP Urugua-í – Arroyo Urugua-í | Misiones | 09/11/2013 | -54.11207 | -25.97775 | 270 | Paraná | 31 | 0.933 |
| RP Urugua-í – A mitad del camino | Misiones | 13/11/2013 | -54.11446 | -25.98951 | 273 | Paraná | 21 | 0.939 |
| RP Urugua-í – Ingreso | Misiones | 14/11/2013 | -53.89194 | -27.15472 | 361 | Paraná | 30 | 0.945 |
| Colanchanga – sitio A | Córdoba | 11/03/2007 | -64.35611 | -31.14611 | 857 | Chaco Serrano | 5 | 0.301 |
| Colanchanga – sitio C1 | Córdoba | 14/02/2007 | -64.35556 | -31.13500 | 954 | Chaco Serrano | 4 | 0.492 |
| Colanchanga – sitio C2 | Córdoba | 10/03/2007 | -64.35556 | -31.13500 | 954 | Chaco Serrano | 6 | 0.617 |
| Colanchanga – sitio C3 | Córdoba | 20/11/2007 | -64.35556 | -31.13500 | 954 | Chaco Serrano | 6 | 0.700 |
| Colanchanga – sitio C4 | Córdoba | 05/12/2007 | -64.35556 | -31.13500 | 954 | Chaco Serrano | 6 | 0.758 |
| Colanchanga – sitio C5 | Córdoba | 06/01/2007 | -64.35556 | -31.13500 | 954 | Chaco Serrano | 5 | 0.799 |
| Los Manantiales – Camino a Pisaditas1 | Córdoba | 20/01/2007 | -64.34139 | -31.16639 | 854 | Chaco Serrano | 2 | 0.829 |
| Los Manantiales – Camino a Pisaditas2 | Córdoba | 21/02/2007 | -64.34139 | -31.16639 | 854 | Chaco Serrano | 3 | 0.851 |
| Los Manantiales – Camino a Pisaditas3 | Córdoba | 11/03/2007 | -64.34139 | -31.16639 | 854 | Chaco Serrano | 11 | 0.868 |
| Los Manantiales – Camino a Pisaditas4 | Córdoba | 18/11/2007 | -64.34139 | -31.16639 | 854 | Chaco Serrano | 6 | 0.881 |
| Los Manantiales – Camino a Pisaditas5 | Córdoba | 16//11/2007 | -64.34139 | -31.16639 | 854 | Chaco Serrano | 4 | 0.892 |
| Los Manantiales – Camino a Pisaditas6 | Córdoba | 06/01/2008 | -64.34139 | -31.16639 | 854 | Chaco Serrano | 2 | 0.900 |
| Los Manantiales – Camino a Pisaditas7 | Córdoba | 15/02/2008 | -64.34139 | -31.16639 | 854 | Chaco Serrano | 2 | 0.907 |
| Los Manantiales – Camino a Pisaditas8 | Córdoba | 01/11/2008 | -64.34139 | -31.16639 | 854 | Chaco Serrano | 1 | 0.913 |
| Colanchanga – sitio B | Córdoba | 21/02/2007 | -64.34139 | -31.14000 | 868 | Chaco Serrano | 4 | 0.918 |
| Camino a Pozos verdes | Córdoba | 19/01/2007 | -64.34444 | -31.15361 | 820 | Chaco Serrano | 4 | 0.923 |
| La Quebrada | Córdoba | 14/02/2007 | -64.34278 | -31.12833 | 978 | Chaco Serrano | 2 | 0.926 |
| Camino a Salsipuedes1 | Córdoba | 21/01/2007 | -64.34333 | -31.12167 | 974 | Chaco Serrano | 7 | 0.930 |
| Camino a Salsipuedes2 | Córdoba | 14/02/2007 | -64.34333 | -31.12167 | 974 | Chaco Serrano | 13 | 0.933 |
| Camino a Salsipuedes3 | Córdoba | 17/11/2007 | -64.34333 | -31.12167 | 974 | Chaco Serrano | 3 | 0.936 |
| Camino a Salsipuedes4 | Córdoba | 05/12/2007 | -64.34333 | -31.12167 | 974 | Chaco Serrano | 5 | 0.939 |
| Camino a Salsipuedes5 | Córdoba | 06/01/2008 | -64.34333 | -31.12167 | 974 | Chaco Serrano | 7 | 0.941 |
| Camino a Salsipuedes6 | Córdoba | 25/02/2008 | -64.34333 | -31.12167 | 974 | Chaco Serrano | 1 | 0.943 |
| Camino a Salsipuedes7 | Córdoba | 15/04/2008 | -64.34333 | -31.12167 | 974 | Chaco Serrano | 6 | 0.946 |
| Colanchanga – sitio A1 | Córdoba | 22/01/2007 | -64.35611 | -31.14611 | 857 | Chaco Serrano | 7 | 0.948 |
| Colanchanga – sitio A2 | Córdoba | 03/12/2007 | -64.35611 | -31.14611 | 857 | Chaco Serrano | 5 | 0.950 |
| Colanchanga – sitio A3 | Córdoba | 18/11/2007 | -64.35611 | -31.14611 | 857 | Chaco Serrano | 4 | 0.952 |
| Colanchanga – sitio A4 | Córdoba | 15/02/2008 | -64.35611 | -31.14611 | 857 | Chaco Serrano | 2 | 0.954 |
| Camino viejo a altas cumbres | Córdoba | 07/01/2011 | -64.71142 | -31.56382 | 1570 | Chaco Serrano | 5 | 0.956 |
| Camino a Nono | Córdoba | 07/01/2011 | -64.95528 | -31.79389 | 865 | Chaco Serrano | 2 | 0.958 |
| Museo Rocsen – a 5km de Nono | Córdoba | 08/01/2011 | -65.00000 | -31.82111 | 965 | Chaco Serrano | 3 | 0.960 |
