# Supplementary material for: Changes in Species Richness and Composition of Tiger Moths (Lepidoptera: Erebidae: Arctiinae) among Three Neotropical Ecoregions
Source: PLoS One. 2016 Sep 28;11(9):e0162661. doi: 10.1371/journal.pone.0162661 (PMC5040457; doi:10.1371/journal.pone.0162661)
Supplement: S2 Table — Presence/Absence of the tiger moth species in each ecoregion. (DOCX) (DOCX) [file pone.0162661.s002.docx]

Hernán M. Beccacece, Sebastián R. Zeballos and Adriana I. Zapata

Changes in species richness and composition of tiger moths (Lepidoptera: Erebidae: Arctiinae) among three neotropical ecoregions

PLOS ONE

**Table S2 List of tiger moth species.** Presence/Absence of tiger moth species in each ecoregion.

| Especies | Yungas | Paraná | Chaco  Serrano |
| --- | --- | --- | --- |
| *Aclytia heber* |  | x | x |
| *Aclytia punctata* |  | x |  |
| *Aclytia terra* |  | x |  |
| *Agaraea nigriostriata* | x |  |  |
| *Agaraea semivitrea* | x | x |  |
| *Agaraea* sp. 1 |  | x |  |
| *Agaraea* sp. 2 |  | x |  |
| *Agaraea* sp. 3 |  | x |  |
| *Agaraea* sp. 4 | x |  |  |
| *Agaraea* sp. 5 | x |  |  |
| *Agaraea uniformis* |  | x |  |
| *Agylla separata* | x | x |  |
| *Agylla* sp. 1 |  | x |  |
| *Agylla* sp. 2 |  | x |  |
| *Agylla* sp. 3 | x |  |  |
| *Agylla* sp. 4 | x |  | x |
| *Amastus antonio* | x |  | x |
| *Amastus formosana* | x |  |  |
| *Amastus* sp. 1 | x |  |  |
| *Amastus steinbachi* | x |  |  |
| *Baritius acuminata* |  | x |  |
| *Baritius nigridorsipeltatus* |  | x |  |
| *Bertholdia albipuncta* |  | x |  |
| *Bertholdia almeidai* |  | x |  |
| *Bertholdia braziliensis* | x |  |  |
| *Bertholdia grisescens* |  | x |  |
| *Bertholdia myosticta* |  | x |  |
| *Bertholdia* sp. 1 |  | x |  |
| *Biturix ochrea* |  | x |  |
| *Biturix rectilinea* | x |  | x |
| *Biturix* sp. 1 | x |  |  |
| *Biturix* sp. 2 |  | x |  |
| *Callisthenia schadei* |  | x |  |
| *Carales astur* | x | x |  |
| *Carales maculicollis* |  | x |  |
| *Chrostosoma annexa* |  | x |  |
| *Chrysocale ferens* | x |  |  |
| *Chrysocale regalis* | x |  |  |
| *Correbidia simonsi* |  | x |  |
| *Cosmosoma auge* |  | x |  |
| *Cosmosoma centralis* |  | x |  |
| *Cosmosoma ignidorsia* |  | x |  |
| *Cosmosoma regia* | x |  |  |
| *Cosmosoma* sp. 1 | x |  |  |
| *Cosmosoma* sp. 2 | x |  |  |
| *Cosmosoma* sp. 3 | x |  |  |
| *Cyanopepla jucunda* |  | x |  |
| *Delphyre brunnea* |  | x |  |
| *Delphyre pyroperas* |  | x |  |
| *Delphyre* sp. 1 |  | x |  |
| *Dycladia lucetius* |  | x |  |
| *Dysschema joiceyi* | x |  |  |
| *Dysschema lucifer* |  | x |  |
| *Dysschema sacrifica* | x | x | x |
| *Dysschema vestalis* |  | x |  |
| *Elysius conjunctus* |  | x |  |
| *Elysius* sp. 1 |  | x |  |
| *Erruca hanga* |  | x |  |
| *Eucereon conffinis* | x |  |  |
| *Eucereon dorsipuncta* |  | x |  |
| *Eucereon pilatti* |  | x |  |
| *Eucereon quadricolor* |  | x |  |
| *Eucereon reticulatum* |  | x |  |
| *Eucereon rosa* |  | x |  |
| *Eucereon rosina* |  | x |  |
| *Eucereon vestalis* |  | x |  |
| *Euchlaenidia neglecta* |  | x | x |
| *Eupseudosoma aberrans* |  | x |  |
| *Eurata strigiventris* | x | x |  |
| *Gardinia* sp. 1 | x |  |  |
| *Graphea paramarmorea* |  | x |  |
| *Gymnelia pavo* | x |  |  |
| *Haematerion sanguinea* |  | x |  |
| *Haematerion* sp. 1 |  | x |  |
| *Haematerion* sp. 2 |  | x |  |
| *Halysidota brasiliensis* |  | x |  |
| *Halysidota nigrilinea* | x |  |  |
| *Halysidota steinbachi* | x |  | x |
| *Halysidota tucumanicola* | x |  | x |
| *Halysitoda brasiliensis* |  | x |  |
| *Horama panthalon viridifusa* | x | x | x |
| *Hyalarctia bertrandi* |  | x |  |
| *Hypercompe abdominalis* | x |  | x |
| *Hypercompe beckeri* | x |  |  |
| *Hypercompe indecisa* |  |  | x |
| *Hypercompe sp.* 1 | x | x | x |
| *Hyperthaema caroei* |  | x |  |
| *Idalus agastus* |  | x |  |
| *Illice* sp. 1 | x | x |  |
| *Illice* sp. 2 | x | x |  |
| *Illice* sp. 3 |  | x |  |
| *Illice* sp. 4 | x | x |  |
| *Illice* sp. 5 |  | x |  |
| *Illice* sp. 6 |  | x |  |
| *Isanthrene* sp. 1 |  | x |  |
| *Ischnocampa lithosioides* |  | x |  |
| *Isia alcumena* | x |  | x |
| *Jorgensenia cunegunda* | x |  |  |
| *Leucanopsis acuta* |  | x |  |
| *Leucanopsis chesteria* |  | x |  |
| *Leucanopsis dinellii* | x |  | x |
| *Leucanopsis leucanina* | x |  |  |
| *Leucanopsis lineata* |  | x |  |
| *Leucanopsis perirrorata* |  | x |  |
| *Leucanopsis polhi* |  | x |  |
| *Leucanopsis* sp. 1 |  | x |  |
| *Leucanopsis* sp. 2 |  | x |  |
| *Leucanopsis* sp. 3 |  | x |  |
| *Leucanopsis* sp. 4 |  | x |  |
| *Leucanopsis* sp. 5 |  | x |  |
| Lithosiini sp. 1 |  | x |  |
| Lithosiini sp. 2 |  | x |  |
| Lithosiini sp. 3 |  | x |  |
| *Lophocampa dinora* | x |  |  |
| *Lophocampa* sp. 1 |  | x |  |
| *Lophocampa texta* |  | x |  |
| *Lophocampa tucumana* | x |  | x |
| *Loxophlebia* sp. 1 |  | x |  |
| *Lycomorphodes* sp. 1 |  | x |  |
| *Lycomorphodes* sp. 2 | x | x |  |
| *Macrocneme leucostigma* |  |  | x |
| *Macrocneme* sp. 1 | x |  |  |
| *Macrocneme* sp. 2 |  | x |  |
| *Mallodeta clavata* |  | x |  |
| *Mazaeras conferta* | x |  |  |
| *Mazaeras yungasensis* | x |  |  |
| *Melese albogrisea* |  | x |  |
| *Melese paranensis* |  | x |  |
| *Mesothen desperata* |  | x |  |
| *Mesothen pyrrhina* |  | x |  |
| *Methysia aenetus* |  | x |  |
| *Methysia* sp. 1 | x |  |  |
| *Munona iridiscens* | x |  |  |
| *Neonerita dorsipuncta* |  | x |  |
| *Neotrichura nigripes* |  | x |  |
| *Neritos cybar* | x |  |  |
| *Nodozana* sp. 1 |  | x |  |
| *Opharus basalis* |  | x |  |
| *Opharus flavimaculata* | x |  |  |
| *Opharus procroides* |  | x |  |
| *Ormetica chrysomelas* |  | x |  |
| *Paracles amarga* | x |  |  |
| *Paracles azollae* |  |  | x |
| *Parahetria triseriata* |  | x |  |
| *Pareuchaetes aurata* | x |  |  |
| *Pareuchaetes* sp. 1 |  | x |  |
| *Pelochyta bicolor* |  | x |  |
| *Pelochyta cinerea* |  | x |  |
| *Phaegoptera depicta* |  | x |  |
| *Phaloe cruenta* | x | x |  |
| *Philoros rubriceps opaca* |  | x | x |
| *Phoenicoprocta analis* |  | x |  |
| *Phoenicoprocta auriflua* | x |  |  |
| *Phoenicoprocta cosquinensis* |  |  | x |
| *Phoenicoprocta* sp. 1 |  | x |  |
| *Phoenicoprocta teda* |  | x |  |
| *Pseudischnocampa nigridorsata* | x |  |  |
| *Pseudohyaleucerea vulnerata* |  | x |  |
| *Pseudosphex* sp. 1 | x |  | x |
| *Rhabdatomis* sp. 1 |  | x |  |
| *Rhynchopyga meisteri* |  | x |  |
| *Rhynchopyga* sp. 1 | x |  |  |
| *Romualdia elongata* |  | x |  |
| *Saurita triangulifera* |  | x |  |
| *Sciopsyche tropica* |  | x |  |
| *Sychesia dryas* |  | x |  |
| *Symphlebia indistincta* |  | x |  |
| *Symphlebia lophocampoides* |  | x |  |
| *Tessella sertata* | x | x | x |
| *Tessellota trifasciata* |  |  | x |
| *Trichromia sardanapalus* |  | x |  |
| *Trichromia* sp. 1 | x |  |  |
| *Trichromia* sp. 2 |  | x |  |
| *Trichromia* sp. 3 | x | x |  |
| *Tricypha nigrescens* |  | x |  |
| *Turuptiana obscura* | x |  |  |
| *Utetheisa ornatrix* | x |  | x |
| *Vianania argentinensis* |  |  | x |
| *Virbia schadei* |  | x |  |
| *Viviennea dolens* |  | x |  |
