# Supplementary material for: Changes in Species Richness and Composition of Tiger Moths (Lepidoptera: Erebidae: Arctiinae) among Three Neotropical Ecoregions
Source: PLoS One. 2016 Sep 28;11(9):e0162661. doi: 10.1371/journal.pone.0162661 (PMC5040457; doi:10.1371/journal.pone.0162661)
Supplement: S3 Table — (DOCX) (DOCX) [file pone.0162661.s003.docx]

Hernán M. Beccacece, Sebastián R. Zeballos and Adriana I. Zapata

Changes in species richness and composition of tiger moths (Lepidoptera: Erebidae: Arctiinae) among three neotropical ecoregions

PLOS ONE

**Table S3 Species observed and non-parametric estimators for incidence data in each ecoregion.** Sobs= Species observed. Estimators: Total of species richness estimated and standard error. Chao2-bc: A Chao 2 bias-corrected for Chao (2005), ICE-1: a modified ICE for highly-heterogeneous cases. Jackknife 1: Estimator that uses the frequency of uniques (Burnham and Overton, 1978); Jackknife 2: Estimators that uses the frequencies of uniques and duplicates (Burnham and Overton, 1978); C hat: Sample coverage index Chao & Jost (2012).

| Non-parametric estimators | Paraná | Yungas | Chaco Serrano |
| --- | --- | --- | --- |
| Sobs | 125 | 63 | 24 |
| Chao2-bc | 138.74±6.87 | 83.11±12.4 | 26.90±3.32 |
| ICE-1 | 144.52 | 80.69±3.6 | 29.14±0.01 |
| Jackknife 1 | 142.47±4.64 | 82.05±5.25 | 29.81±2.57 |
| Jackknife 2 | 155.61 | 93.27 | 31.8 |
| C. hat | 0.945 | 0.920 | 0.960 |
