# Supplementary material for: Changes in Species Richness and Composition of Tiger Moths (Lepidoptera: Erebidae: Arctiinae) among Three Neotropical Ecoregions
Source: PLoS One. 2016 Sep 28;11(9):e0162661. doi: 10.1371/journal.pone.0162661 (PMC5040457; doi:10.1371/journal.pone.0162661)
Supplement: S4 Table — Evaluated by Chao-Jaccard distance matrix and pairwise comparison between ecoregions. (DOCX) (DOCX) [file pone.0162661.s004.docx]

Hernán M. Beccacece, Sebastián R. Zeballos and Adriana I. Zapata

Changes in species richness and composition of tiger moths (Lepidoptera: Erebidae: Arctiinae) among three neotropical ecoregions

PLOS ONE

**Table S4 A*donis* test partitioning variation on species composition evaluated by Chao-Jaccard distance matrix and pairwise comparison between ecoregions.**

| Source of variation | df | SS | MS | F | R^2^ | p |
| --- | --- | --- | --- | --- | --- | --- |
| Ecoregion | **2** | **7.2697** | **3.6348** | **12.608** | **0.27** | **0.0001** |
| Sites | **68** | **19.6049** | **0.2883** |  | **0.73** |  |
| Total | **70** | **26.8745** |  |  | **1** |  |

| Pairwise comparison | df | F | p (perm) |
| --- | --- | --- | --- |
| Yungas – Paraná | **2, 68** | **31.94** | **0.0001** |
| Yungas – Chaco Serrano | **1, 50** | **14.821** | **0.0001** |
| Paraná – Chaco Serrano | **1, 48** | **26.033** | **0.0001** |
